# Casein Kinase I**γ**2 Impairs Fibroblasts Actin Stress Fibers Formation and Delays Cell Cycle Progression in G1

**DOI:** 10.1155/2012/684684

**Published:** 2012-03-07

**Authors:** Mathieu Latreille, Afnan Abu-Thuraia, Rossella Oliva, Dongmei Zuo, Louise Larose

**Affiliations:** Polypeptide Laboratory, Department of Medicine, McGill University, Montreal, QC, Canada H3A 2B2

## Abstract

Actin cytoskeleton remodeling is under the regulation of multiple proteins with various activities. Here, we demonstrate that the **γ**2 isoform of Casein Kinase I (CKI**γ**2) is part of a novel molecular path regulating the formation of actin stress fibers. We show that overexpression of CKI**γ**2 in fibroblasts alters cell morphology by impairing actin stress fibers formation. We demonstrate that this is concomitant with increased phosphorylation of the CDK inhibitor p27^Kip^ and lower levels of activated RhoA, and is dependent on CKI**γ**2 catalytic activity. Moreover, we report that roscovitine, a potent inhibitor of cyclin-dependent kinases, including Cdk5, decreases p27^Kip^ protein levels and restores actin stress fibers formation in CKI**γ**2 overexpressing cells, suggesting the existence of a CKI**γ**2-Cdk5-p27^Kip^-RhoA pathway in regulating actin remodeling. On the other hand, we also show that in a manner independent of its catalytic activity, CKI**γ**2 delays cell cycle progression through G1. Collectively our findings reveal that CKI**γ**2 is a novel player in the control of actin cytoskeleton dynamics and cell proliferation.

## 1. Introduction

The Rho family of GTPases comprising RhoA, Rac1, and Cdc42 regulates the organization of the cytoskeleton in eukaryotic cells [1]. These proteins cycle between an active GTP-bound and inactive GDP-bound state through the action of GTPase exchange factors (GEFs) and GTPase activating proteins (GAPs) [2]. Once activated, RhoA regulates actin stress fibers formation [3], while Rac1 triggers the assembly of actin in lamellipodia and membrane ruffles [4] and Cdc42 induces filopodial extensions [5] at the leading edge of the cell. Over the years, Rho GTPases were found to be crucial regulators of actin remodeling involved in a great deal of normal cellular functions, including cell migration and adhesion, cell cycle progression, and membrane trafficking [6]. In addition, Rho GTPases contribute to pathological conditions, particularly to cancer initiation and metastasis by controlling cell proliferation, migration, and adhesion during oncogenic transformation [7–9].

 Accumulating evidence suggests that Rho GTPases are regulated at least in part by the cyclin-dependent kinase inhibitors (CDKIs) p21^Waf/Cip^, p27^Kip1^, and p57^Kip2^ through different mechanisms. As example, p27^Kip1^, which depends on its abundance and nuclear localization to inhibit the cyclin-dependent kinases (CDKs), inhibits RhoA activation in a cell-cycle independent manner, thereby modulates actin dynamics [10]. In fact, p27^Kip1^ phosphorylation at Ser10 increases its stability and cytoplasmic localization [11, 12], where it binds to and inhibits RhoA by interfering with the interaction between RhoA and its activating GEFs [10]. Among protein kinases that regulate p27^Kip1^, cyclin-dependent kinase 5 (Cdk5), also known as a regulator of actin dynamics, was found to stabilize p27^Kip1^ through phosphorylation of p27^Kip1^ at Ser10 in cortical neurons [13]. However, whether Cdk5 possesses similar activity in nonneuronal cells remains to be determined.

 Casein kinase I (CKI) encompass a large family of Ser/Thr protein kinases encoded by separate genes and several splice variants. The 7 mammalian CKI isoforms identified so far, namely, *α*, *β*, *γ*1–3, *δ*, and *ε*, share high degree of identity within their kinase domain, but differ significantly in the length and amino acid composition of their N- and C-termini [14]. Overall, CKIs are conserved throughout evolution and involved in diverse cellular functions [15]. CKI*α*, *δ*, and *ε* involved in vesicular trafficking [16–18] are also implicated in canonical Wnt signaling, but with distinct role [19]. CKI*δ* transduces specific centrosome functions [20], but, like CKI*ε*, it also contributes to the regulation of the circadian rhythm [21, 22], apoptosis [23], and neuronal neurite outgrowth [24]. Interestingly, among the CKI family, the closely related CKI*γ* proteins (CKI*γ*1, 2, and 3) are unique in carrying C-terminal lipid modification motif that is believed to anchor them at the plasma membrane [25, 26]. In agreement with CKI*γ* plasma membrane localization, expression of the *Xenopus tropicalis* CKI*γ* in vertebrates and *Drosophila* cells has been implicated in transducing early signaling events of LRP6, a cell surface membrane receptor involved in Wnt signaling [25]. However, very little is known regarding the function of individual mammalian CKI*γ* isoforms. Previously, we reported that the Src homology (SH) domain-containing adaptor protein Nck directly interacts with CKI*γ*2 through two of its SH3 domains [27], while we determined that a proline rich motif (P^343^DVPSQPR^352^) unique to the C-terminal noncatalytic tail of CKI*γ*2 is mediating binding of Nck (unpublished data). Given that Nck transduces signals from membrane receptor protein tyrosine kinases to effectors regulating crucial biological cellular responses such as actin cytoskeletal reorganization and cell proliferation, we further investigated CKI*γ*2 function in mammalian cells.

In this study, we provide evidence that the kinase activity is required for CKI*γ*2 to regulate actin cytoskeleton remodeling through its ability to downregulate RhoA proteins and signaling via the activation of the Cdk5-p27^Kip1^ pathway. In addition, our findings also reveal that in a manner independent of its catalytic activity, CKI*γ*2 also regulates cell proliferation.

## 2. Materials and Methods

### 2.1. CKI*γ* Constructs

The mouse CKI*γ*1, 2, and 3 full length cDNAs were subcloned downstream of a Kozak sequence and in frame with a HA epitope sequence into the mammalian expression vector pZeoSV2 (Invitrogen). A kinase deficient (KD) CKI*γ*2 full length cDNA was generated by introducing a point mutation (K^75^R) in the ATP-binding site. A cDNA (1–1020 nts) encompassing the kinase domain, but lacking the C-terminal extension of CKI*γ*2 (Δ C-term), was generated by PCR using appropriate specific primers and further subcloned into pZeoSV2 as reported above. All constructs were fully sequenced to confirm their identity and to ensure that no unwanted mutation had been introduced during their creation.

### 2.2. Stable Cell Lines of Fibroblast Overexpressing CKI*γ*2

Rat-2 fibroblasts were cultured in DMEM (Dulbecco's modified Eagle's medium; Life Technologies, Inc) supplemented with 2 mM L-glutamine, 45 mM sodium bicarbonate, and 10% FBS at 37°C, in a humidified atmosphere of 95% air and 5% CO_2_. Using calcium phosphate precipitation, fibroblasts were transfected with indicated expression plasmids. Upon selection in medium containing high concentration of zeocin (500 *μ*g/mL) or G418 (400 *μ*g/mL) for cells transfected, respectively, with pZeoSV2 or pcDNA 3.1, individual clones were isolated, grown, and analyzed for expected proteins expression. Positive clones were propagated under the same conditions, except that 50 *μ*g/mL zeocin or 40 *μ*g/mL G418 was added to the culture medium. For fibroblasts transfected with the empty pZeoSV2 plasmid, instead of individual clones following zeocin selection procedure, a pool of resistant cells was propagated and used as control.

### 2.3. Cell Culture and Transient Transfection

Rat-2 and HaCaT cells were grown in DMEM and HepG2 cells in Minimum Essential Medium Alpha Medium (MEM) (Invitrogen) supplemented with antibiotic/antimycotic (Invitrogen) and 10% heat-inactivated fetal bovine serum (FBS) (Invitrogen) at 37°C in 5% CO_2_/95% O_2_. For CKI*γ*1, 2, and 3 transient expression into Rat-2 cells, cells plated at 80% confluency in 60 mm dishes were transiently transfected with indicated expression plasmids using Lipofectamine-Plus reagent (Invitrogen) according to the manufacturer's instructions.

### 2.4. SiRNA Transfection

Human CK1*γ*2 siRNAs targeting two independent coding regions (R1 and R2) were purchased from Integrated DNA technologies (IDT) R1(5′-GCACCUGGAGUACCGGUUC-3′) and R2(5′-GCGCUACAUGAGCAUCAAC-3′). Scrambled siRNA obtained also from IDT was used as control. HepG2 and HaCaT cells were transiently transfected with indicated siRNA using Lipofectamine RNAiMAX reagent (Invitrogen) according to the manufacturer's instructions. Briefly, 300 *μ*mol of siRNA was added to 500 *μ*L of Opti-MEM I Medium without serum (Invitrogen) in 6-well plates and mixed gently. 5 *μ*L of Lipofectamine RNAiMAX reagent was added to each well containing diluted siRNAs, mixed gently, and incubated at room temperature for 20 min. In the meantime, cells were harvested, counted, and diluted at 200 000 cells/mL in MEM media without antibiotics. Then, 2.5 mL of cells suspension (i.e., 500 000 cells/well) were added to each well and mixed gently, making the final siRNA concentration at 100 nM. The cells were further incubated at 37°C for 48–72 hours.

### 2.5. Antibodies, Immunoprecipitation, and Western Blots

To immunoprecipitate HA-tagged CKI*γ*2, we used the commercial HA F-7 antibody (Santa Cruz). For western blot analysis, the following antibodies were used: HA Y-11 (Santa Cruz); p53 FL-393 (Santa Cruz), Nck 1794 (in house [27]), p21^Cip1^ C-10 (Santa Cruz);  p27^Waf1^ C-19 (Santa Cruz) and RhoA F-1 (Santa Cruz). To detect CKI*γ*2, we generated a rabbit polyclonal antibody using a KHL-coupled CKI*γ*2 peptide encompassing aa 331–354 as antigen. In general, cells were lysed in lysis buffer (50 mM Hepes, pH 7.5, 150 mM NaCl, 10% Glycerol, 1% Triton X-100, 1.5 mM MgCl_2_, 1 mM EGTA, 10 mM sodium pyrophosphate, 10 mM sodium fluoride) supplemented with 2 *μ*g/mL leupeptin and aprotinin as well as with 1 mM phenyl-methylsulfonyl fluoride (PMSF) and 200 *μ*M activated sodium orthovanadate. Clarified cell lysates were normalized to equal protein concentrations with the lysis buffer and protein immunoprecipitations performed using appropriate antibodies. Immune complexes were subsequently collected with Protein A-Agarose (SantaCruz), and, after several washes with the lysis buffer, proteins were eluted in Laemmli buffer [28], boiled, and subjected to SDS-PAGE. Western Blots were performed as previously described [29] using chemiluminescence (ECL Plus, GE Healthcare, UK). When mentioned, equal amounts of total cell proteins were subjected to SDS-PAGE and subjected to Western Blot analysis following the same protocol.

### 2.6. In  Vitro Kinase Assays

Immunoprecipitated proteins immobilized on Protein A beads or recombinant GST fusion proteins were washed five times with lysis buffer and three times with the kinase buffer before being divided in two aliquots, which were, respectively, subjected to *in vitro* kinase assay and immunoblot. For CKI activity, the kinase buffer was composed of 20 mM Hepes, pH 7.5, 1 mM dithiothreitol (DTT), 5 mM MgCl_2_, 10 mM *β*-glycerophosphate, and 5 *μ*g of *α*-casein as exogenous substrate. For all assays, following a preincubation at 30°C for 5 min, the reactions were initiated by adding [*γ*-^32^P]-ATP (50 *μ*M, 5–10 *μ*Ci) (DuPont, NEN) and further incubated for 20 min at 30°C. The reactions were stopped by adding Laemmli buffer, boiled, subjected to SDS-PAGE and then to autoradiography. Phosphorylation of exogenous substrates was analyzed by densitometry (Imaging Densitometer, Model GS-800, BioRad). To assess whether CKI*γ*2 phosphorylates RhoA *in vitro*, 200 ng of purified recombinant GSTCKI*γ*2 full length (FL) or truncated of its C-terminal (Δ C-term) were incubated with 1 *μ*g of purified recombinant RhoA as reported above.

### 2.7. Cell Proliferation

Proliferation of stable fibroblast cell lines was evaluated by counting the number of cells, different times after plating. Cells were seeded at 5 × 10^3^ cells/60 mm plate, in triplicate for each time points and cell lines. On days 3, 5, and 7 after plating, the cells were trypsinized and counted using a hemocytometer.

### 2.8. ^3^H-Thymidine Incorporation

Cells were plated at 2 × 10^4^cells/well in 24 wells plates and grown for 24 hours in DMEM containing 10% FBS. The next day, the cells were starved for 36 hours in DMEM supplemented with 0.1% BSA. At the end of the starvation period, the medium was replaced by fresh starving medium with or without FBS at 2.5% or PDGF at 25 ng/mL and the cells incubated for an additional 24 hours. During the last 8 hours of stimulation, 0.5 *μ*Ci of ^3^H-Thymidine was added. Thymidine incorporation was stopped by replacing the medium by cold TCA (10%) and further incubation at 4°C. Precipitated material was then solubilized in 0.3 N NaOH and incorporated ^3^H-Thymidine counted by liquid scintillation using a LKB 1219 Rack Beta Liquid scintillation Counter.

### 2.9. DNA Laddering

Following washes with PBS, serum growing cells in culture dishes were directly lyzed in 0.5 mL of DNAzol genomic isolation reagent (Molecular Research Center, Inc., Cincinnati, OH). The resulting lysates were subjected to repeated pipetting and DNA precipitation performed by adding 0.25 mL of 100% ethanol. Samples were mixed by inverting the tubes 5–8 times and kept at room temperature for 3 min. Precipitated DNA was then spooled using a pipette tip, washed twice in 70% ethanol, and dissolved in water. Samples of total DNA were separated on 1.8% agarose gel and stained with ethidium bromide. As positive control, primary rat thymocytes maintained in culture in DMEM supplemented with 10% FBS were treated with 10 *μ*g/mL of anisomycin for 24 hours. Thymocytes were collected by centrifugation, washed with PBS and genomic DNA prepared as described above.

### 2.10. Cell Cycle Analysis

For flow cytometry analysis (FACS), 1 × 10^6^ of serum growing cells were collected, fixed in 70% ethanol following incubation for 15 min on ice and storage for at least 1 hour at −20°C. Fixed cells were washed in cold PBS, and stained with propidium iodide (PI, Sigma) using a solution containing 50 *μ*g/mL of PI and 10 *μ*g/mL of RNAse in PBS at 37°C for 30 min. Quantification of cell populations in different phases of the cell cycle was determined using the Cell Quest software (Becton Dickinson, CA).

### 2.11. Cell Morphology and Actin Staining

Cells plated on coverslips were rinsed with PBS before being fixed for 10 min at room temperature in 4% formaldehyde/PBS. Following fixation, coverslips were rinsed with PBS and the cells permeabilized in 0.2% Triton X-100/PBS for 5 min at room temperature. For filamentous actin staining, cells were incubated with rhodamine-conjugated phalloidin (0.1 *μ*g/mL; Sigma, Oakville, ON. Canada) or phalloidin-coupled to Alexa Fluor 488Fluor for 30–60 min at room temperature. For HA-staining, we used the commercially available anti-HA 12CA5 (Roche Apllied Science). Coverslips were washed with PBS and water prior to being mounted with Mowiol and examined on a Zeiss Axiovert 200 microscope at 40X or 63X using Zeiss oil immersion. Fluorescence images were subsequently captured using a digital camera (DVC) and analyzed with Northern Eclipse software (Empix Imaging Inc.). Images were transferred to Adobe Photoshop and assembled with PowerPoint.

### 2.12. Rho Activation Assays

Essentially, levels of activated RhoA (RhoA-GTP) were assessed using the Rho activation kit purchased from Millipore (cat. no. 17–294). Briefly, serum growing fibroblasts (R2Zeo and Z23), about 70% confluent, were transiently transfected with a vector-encoding Myc-tagged RhoA (100 ng) using Lipofectamine Plus (Invitrogen). Cells lysates prepared 16 hours after transfection were mixed with 60 *μ*g of recombinant GST-Rhotekin Rho binding domain previously isolated on beads. Following 45 min at 4°C, beads were washed three times, boiled in Laemmli sample buffer, and bound proteins separated on a 12% SDS-polyacrylamide gel. Levels of Myc-tagged RhoA proteins bound to the fusion protein or present in the whole cell lysates were evaluated by western blotting with a rabbit polyclonal anti-Rho antibody (RhoA, B, and C) provided with the kit and ECL Plus detection as reported above.

### 2.13. Cells Stimulation

Cells (6 × 10^4^) were plated on coverslips 24 hours prior to be serum starved for 24 hours in DMEM/0.1% BSA and subsequently treated with 50 ng/mL of lysophosphatidic acid (LPA, Sigma) for 30 min at 37°C or overnight. For roscovitine experiments, we treated the cells overnight with 25 *μ*M roscovitine (Sigma). Control cells were exposed to equivalent volume of vehicle. Cells were then washed, stained for filamentous actin using phalloidin and mounted for immunofluorescence microscopy or processed for western blot analysis as previously described.

## 3. Results

### 3.1. CKI*γ*2 Overexpression in Fibroblasts Alters Cell Morphology and Inhibits Actin Stress Fibers Formation in a Kinase-Dependent Manner

**Figure 1:**
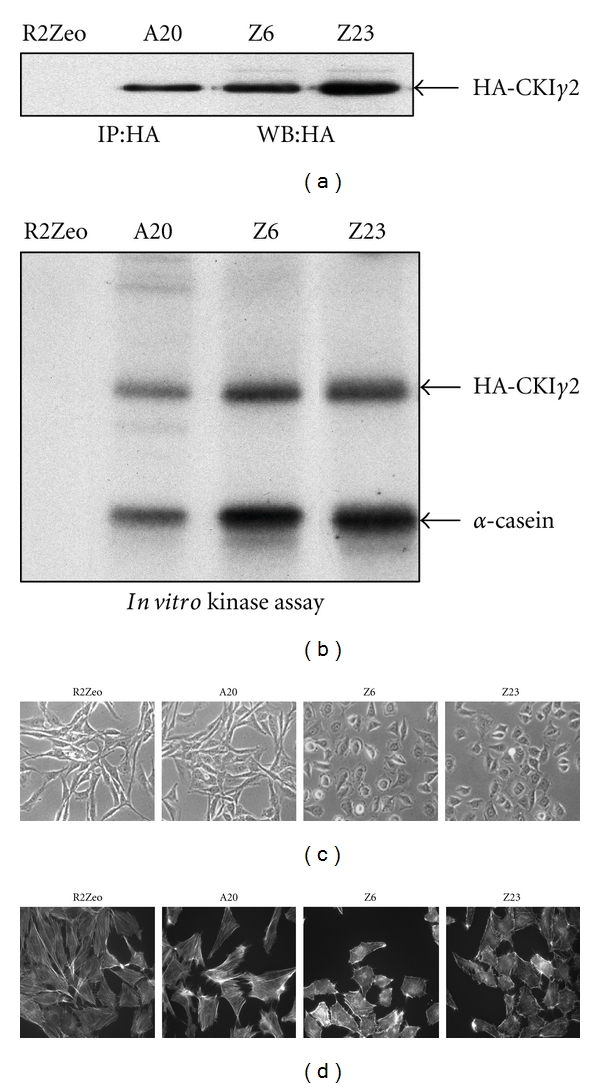
CKI*γ*2 overexpression in fibroblasts alters cell morphology and actin stress fibers formation. (a) Isolated clones of fibroblasts stably transfected with a plasmid encoding HA-tagged-CKI*γ*2 (A20, Z6, and Z23) or pool of stable cells transfected with an empty plasmid (R2Zeo) were analyzed for HA-CKI*γ*2 expression by Western Blot (WB) on HA immunoprecipitates (IP). (b) CKI*γ*2 activity determined on HA immunoprecipitates in *in vitro* kinase assays using phosphorylation of exogenous *α*-casein. (c) Indicated serum growing cells were analyzed for cell morphology by phase contrast microscopy (40X) and (d) actin organization using rhodamine-conjugated phalloidin staining (63X). R2Zeo: control; A20, Z6, and Z23: clones overexpressing increasing levels of CKI*γ*2.

To investigate the role of CKI*γ*2 in mammalian cells, we generated fibroblasts that stably overexpress CKI*γ*2 by transfecting a plasmid encoding N-terminal HA-tagged wild-type CKI*γ*2 [29]. Fibroblasts transfected with an empty plasmid are considered as control. We selected a pool of empty plasmid transfected cells (R2Zeo) as control and three independent clones expressing different levels of the 50–55 kda HACKI*γ*2 protein (A20 < Z6 < Z23) to further study (Figure 1(a)). We demonstrated the activity of HA-CKI*γ*2 by performing *in vitro* kinase assays on HA immunoprecipitates (IP) using *α*-casein as exogenous substrate (Figure 1(b)). Visual examination of these cells foremost revealed that fibroblasts overexpressing higher levels of CKI*γ*2 (Z6 and Z23) presented marked change of morphology when compared with fibroblasts overexpressing lower levels of CKI*γ*2 (A20) or mock-transfected fibroblasts (R2Zeo) (Figure 1(c)). We observed that cells harboring higher levels of CKI*γ*2 (Z6 and Z23) lost their fibroblastic elongated shape to acquire a more rounded morphology. Actin staining with phalloidin demonstrated that the rounded shrunken morphology of these cells (Z6 and Z23) is associated with a drastic decrease in actin stress fibers (Figure 1(d)).

**Figure 2:**
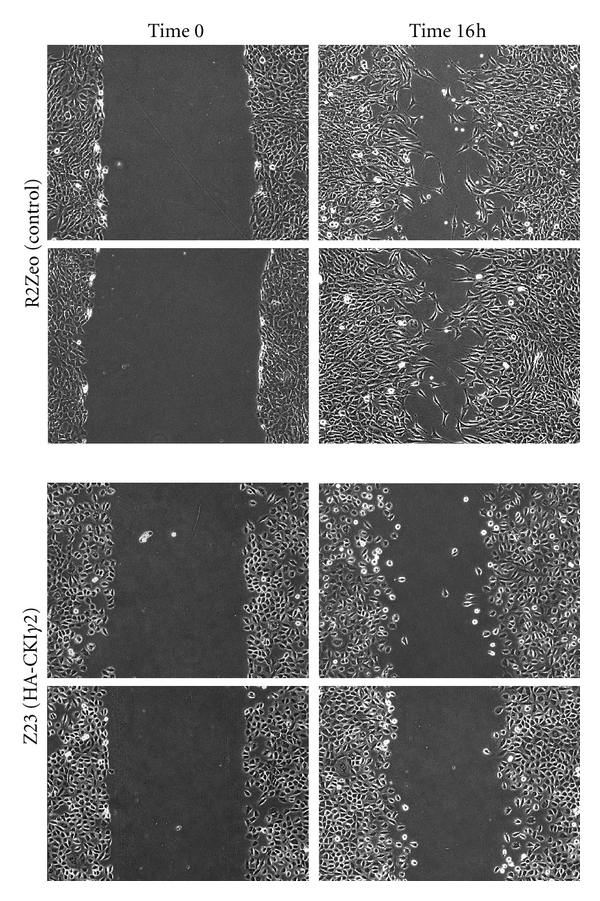
CKI*γ*2 overexpression in fibroblasts impairs cell motility. Migration of serum starved control (R2Zeo) and HA-CKI*γ*2 overexpressing fibroblasts (Z23) were evaluated in wound healing assays. Pictures (10X) from the same area were taken at time 0 and 16 hours after the wound.

 To assess whether loss of actin stress fibers in fibroblasts overexpressing CKI*γ*2 affects cell motility, we compared the migratory activity of fibroblasts overexpressing CKI*γ*2 (Z23) with control fibroblasts (R2Zeo) using *in vitro* wound healing assays. To ensure that cells in the wounded area result from cell motility, rather than proliferation, fibroblasts were deprived from serum for 24 hours prior to performing the wound. As shown in Figure 2, fibroblasts that overexpress CKI*γ*2 did not migrate and fill the wounded area at a rate comparable to control fibroblasts. Altogether, these observations indicate that overexpression of CKI*γ*2 in fibroblasts induces dissolution of actin stress fibers and impairs cell motility *in vitro*.

**Figure 3:**
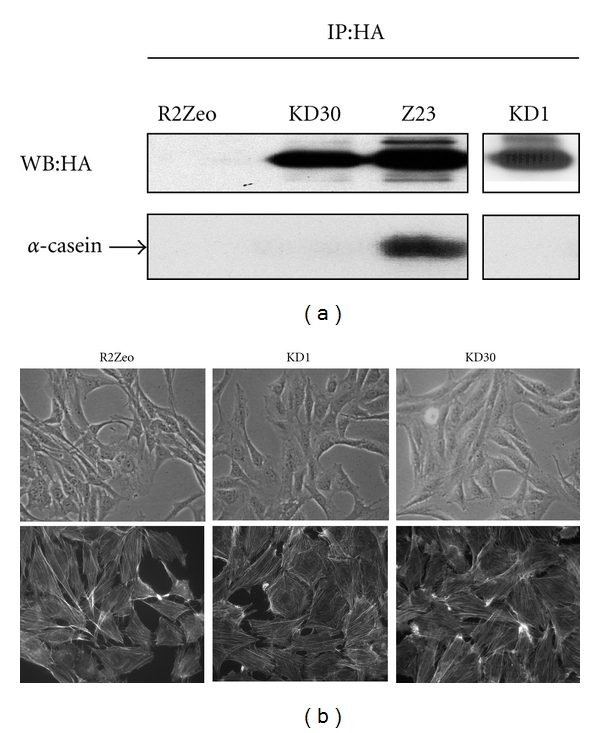
The catalytic activity of CKI*γ*2 is required to induce change in cell morphology and actin stress fibers in fibroblasts. (a) Isolated clones of fibroblasts stably transfected with a plasmid encoding HA-tagged wild-type CKI*γ*2 (Z23), HA-tagged kinase deficient CKI*γ*2 (KD1 and KD30), or pool of stable fibroblasts transfected with an empty plasmid (R2Zeo) were analyzed for HA-CKI*γ*2 expression by HA Western Blot (WB) on HA immunoprecipitates (IP) and CKI*γ*2 activity as determined on HA immunoprecipitates from equal amounts of protein normalized cell lysates and *in vitro* kinase assays using *α*-casein as substrate. KD1 and KD30: clones that overexpress kinase deficient CKI*γ*2 at the same levels as cells stably overexpressing wild-type CKI*γ*2 (Z23). (b) Morphology of serum growing cells was visualized by phase contrast microscopy (40X) (upper panels) and actin organization by actin staining with rhodamine-conjugated phalloidin (63X) (lower panels).

We next investigate whether the kinase activity is required for CKI*γ*2 to inhibit the formation of actin stress fibers. For this, we generated two independent clones of fibroblasts stably overexpressing a kinase deficient form of CKI*γ*2 (KD1, KD30) at levels almost comparable to wild-type CKI*γ*2 levels detected in the Z23 cell line (Figure 3(a), upper panel). As expected, CKI*γ*2 KD (K75R) is devoid of catalytic activity as shown by the absence of *α*-casein phosphorylation in HA-immunoprecipitated CKI*γ*2 KD in *in vitro* kinase assays (Figure 3(a), lower panel). However, we observed similar to control cells (R2Zeo) morphology and levels of actin stress fibers organization in fibroblasts overexpressing kinase deficient CKI*γ*2 (KD) (Figure 3(b)). This demonstrates that the kinase activity of CKI*γ*2 is required for the inhibition of actin stress fibers formation.

**Figure 4:**
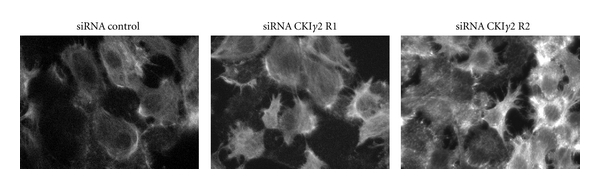
Increased actin stress fibers in HaCaT cells transiently transfected with hCKI*γ*2 siRNAs. HaCaT cells transiently transfected with siRNA control or siRNAs targeting two independent coding regions of hCKI*γ*2 (R1, R2) were subjected to actin staining using phalloidin-coupled to AlexaFluor 488. Pictures were taken at 63X.

 To demonstrate that the regulation of actin stress fiber formation by CKI*γ*2 occurs not only in overexpressing conditions, we assessed actin stress fibers in HaCaT human keratinocytes transiently transfected with two siRNAs (R1, R2) derived from short hairpin-type RNA constructs targeting independent coding regions of hCKI*γ*2 that have been reported to effectively downregulate CKI*γ*2 in these cells [30]. As shown in Figure 4, HaCaT cells treated with CKI*γ*2 siRNAs substantially present increased formation of stress fibers, supporting a physiological role for CKI*γ*2 in regulating actin cytoskeleton reorganization.

### 3.2. CKI*γ*2 Overexpression in Fibroblasts Decreases RhoA Protein and RhoA-GTP Levels

**Figure 5:**
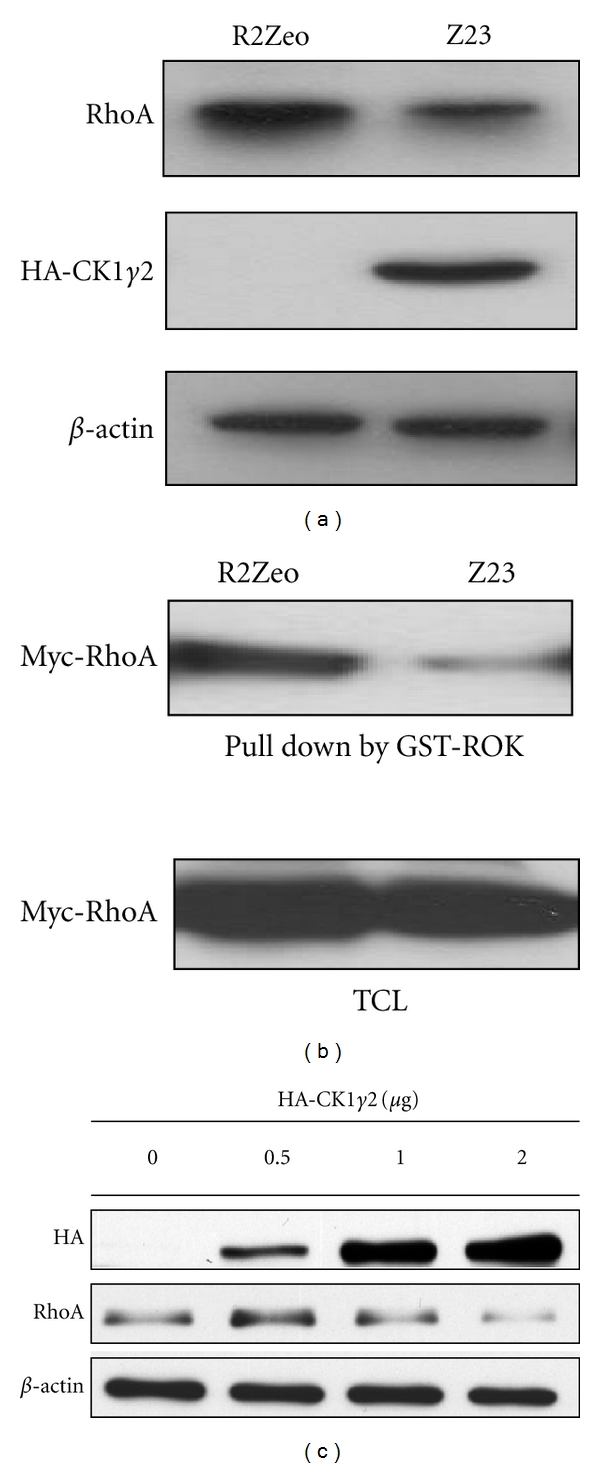
Overexpression of CKI*γ*2 in fibroblasts reduces the levels of RhoA protein and RhoA activation. (a) Fibroblasts stably overexpressing CKI*γ*2 (Z23) and control fibroblasts (R2Zeo) were analyzed for RhoA protein expression levels by Western Blot using equivalent amount of total cell lysate proteins. (b) Levels of activated Myc-RhoA (Myc-RhoA-GTP) were assessed following transient expression of MycRhoA in serum growing fibroblasts control (R2Zeo) or overexpressing CKI*γ*2 (Z23) using pull down of equivalent amount of total cell lysate proteins with a GST fusion protein encoding the Rho-binding domain of Rhotekin. (c) Total cell lysates from fibroblasts transiently transfected with increasing amount of plasmid encoding HA-CKI*γ*2 were subjected to Western Blot analysis with indicated antibodies.

Formation of actin stress fibers is under the control of the small GTPases Rho [3]; therefore, we first compared the levels of RhoA protein in fibroblasts overexpressing CKI*γ*2 with control fibroblasts (Figure 5(a)). Interestingly, we found that overexpression of CKI*γ*2 results in decreased levels of the RhoA proteins, suggesting that dissolution of actin stress fibers in CKI*γ*2 overexpressing fibroblasts might be due to low levels of RhoA proteins that yield to nonefficient RhoA signaling activity. To further investigate this point, we expressed Myc-RhoA in fibroblasts overexpressing or not CKI*γ*2 and determined the levels of active Myc-RhoA-GTP by measuring the amount of Myc-RhoA proteins bound by a GST fusion protein encoding the Rho-binding domain of Rhotekin. Consistent with decreased actin stress fibers and lower RhoA protein levels in fibroblasts overexpressing CKI*γ*2, we found lower levels of activated RhoA (Myc-RhoA-GTP) as well as total Myc-RhoA in cells overexpressing higher levels of CKI*γ*2 (Figure 5(b)). To further support that increased expression of CKI*γ*2 downregulates RhoA protein levels, we transiently transfected Rat-2 fibroblast with increasing amounts of plasmid encoding HA-CKI*γ*2 and assessed expression levels of HA-CKI*γ*2 and RhoA in total cell lysates by western blotting. In agreement with decreased levels of RhoA protein in fibroblasts overexpressing high levels of CKI*γ*2 (Z23), transient expression of high levels of CKI*γ*2 leads to lower levels of RhoA protein (Figure 5(c)). Altogether, these data suggest that CKI*γ*2 contributes to lowering the expression or enhancing the degradation of RhoA and this could result in attenuated RhoA signaling.

**Figure 6:**
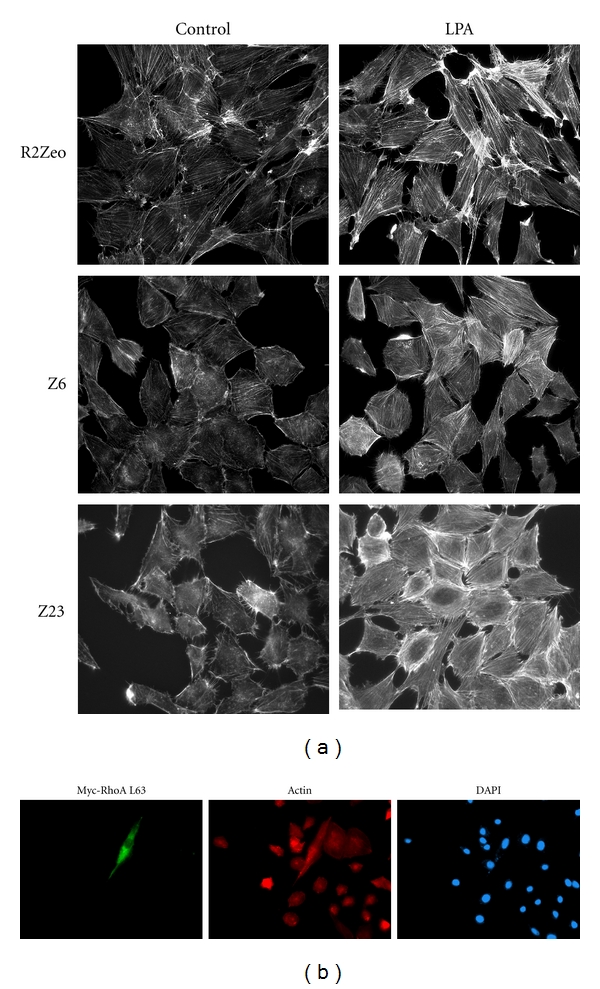
LPA stimulation and activated RhoA rescue actin stress fibers formation in fibroblasts overexpressing CKI*γ*2. (a) Actin staining following LPA stimulation (50 ng/mL, 10–30 minutes, and 37°C) in serum-starved fibroblasts control (R2Zeo) or stably overexpressing CKI*γ*2 (Z6 and Z23). Pictures were taken at 63X. (b) Myc, Actin, and DAPI staining of CKI*γ*2 overexpressing fibroblasts (Z23) transiently transfected with a cDNA encoding a constitutively activate form of RhoA (Myc-RhoA L63). Pictures were taken at 40X.

To determine whether fibroblasts overexpressing CKI*γ*2 can still be challenged by external stimuli to build up actin stress fibers, we treated these cells with the serum-borne phospholipid lysophosphatidic acid (LPA), a G-protein-coupled receptor agonist which regulates the assembly of actin stress fibers through the activation of RhoA [31]. Actin staining of fibroblasts expressing high levels of HA-CKI*γ*2 in response to LPA stimulation at 50 ng/mL for 10–30 min revealed that, in all conditions, LPA treatment results in formation of actin stress fibers (Figure 6(a)). Finally, actin stress fibers could be rescued by expressing a constitutively active RhoA (RhoAL63) in fibroblasts overexpressing CKI*γ*2. Altogether, these data suggest that signaling downstream of RhoA is intact in fibroblasts overexpressing CKI*γ*2 and it also could be efficiently challenged to lead to the formation of actin stress fibers (Figure 6(b)). Overall, our observations provide strong evidence supporting that CKI*γ*2-mediated inhibition of RhoA-dependent formation of actin stress fibers is reversible and could result from impaired expression and activation of the GTPases Rho.

### 3.3. RhoA Is Not Phosphorylated by CKI*γ*2 In Vitro

**Figure 7:**
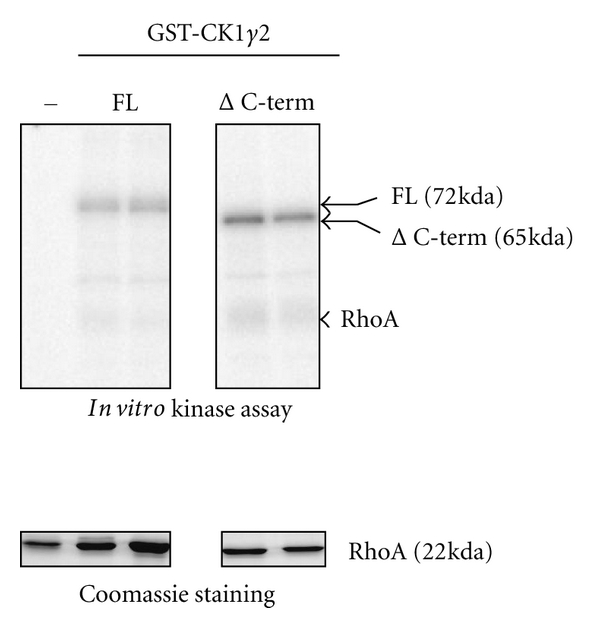
RhoA is not phosphorylated by CKI*γ*2 *in vitro*. Recombinant GST-CKI*γ*2 full length (FL) or C-terminal truncated (Δ C-term) (200 ng) purified on beads was incubated with recombinant RhoA (1 *μ*g) in *in vitro* kinase assays. Incorporation of ^32^P was revealed by autoradiography of the kinase reactions resolved on SDS-PAGE. Respective proteins are indicated, and equivalent amount of RhoA used in the assays was revealed by Coomassie blue staining.

As serine phosphorylation of Rho proteins negatively regulates their activity, we determined whether CKI*γ*2 could directly phosphorylate RhoA *in vitro*. For this, we incubated GST fusion protein encoding CKI*γ*2 full length (FL) or truncated with its noncatalytic C-terminal domain deleted (Δ C-term), with recombinant RhoA in presence of [*γ*-^32^P] ATP and assessed ^32^P labeling of RhoA upon SDS-PAGE and autoradiography. As shown in Figure 7, CKI*γ*2 full length and CKI*γ*2 deleted of its C-terminal domain autophosphorylate *in vitro*, suggesting that these are active protein kinases. In contrast, RhoA was not phosphorylated by either GST-CKI*γ*2 constructs, suggesting that *in vivo* CK*γ*2 does not induce actin stress fibers disassembly by directly phosphorylating and inhibiting RhoA.

### 3.4. CKI*γ*2 Overexpression in Fibroblasts Inhibits Cell Proliferation and Delays Cell Cycle Progression in G1

**Figure 8:**
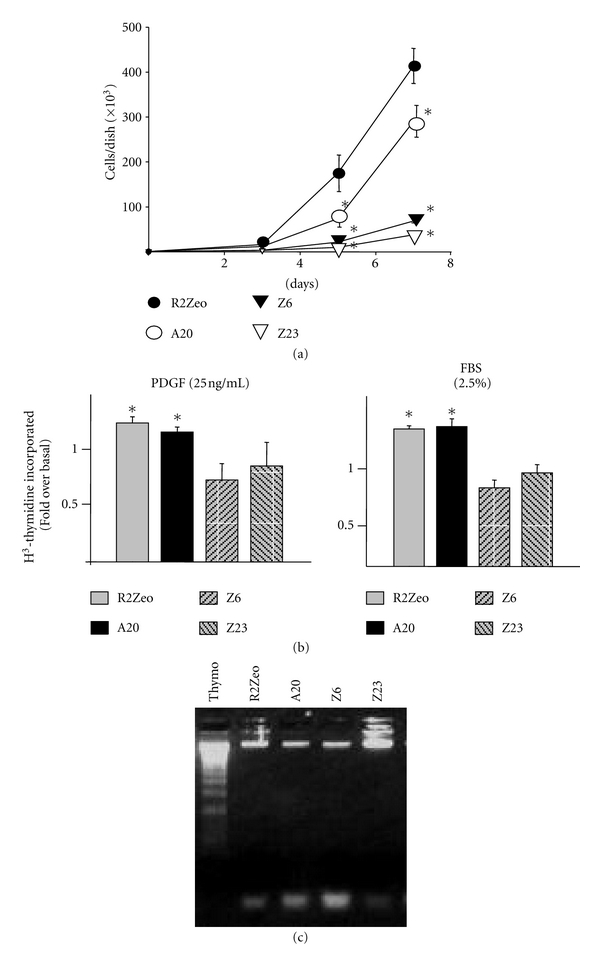
CKI*γ*2 overexpression in fibroblasts reduces cell proliferation. (a) Cell proliferation was examined by seeding each cell line in triplicate at 3 × 10^5^ cells/60 mm plate on day 0. On days 3, 5, and 7, cells were trypsinized and counted. Results are expressed as the average ± SEM of three independent experiments and for Z6 and Z23, the symbols include the SEM. *Significantly different at least at *P* < 0.01 as determined by Student's *t*-test when compared with R2Zeo cells. (b) Incorporation of ^3^H-thymidine in response to PDGF (25 ng/mL) or serum (2.5% FBS) stimulation was performed in indicated cell lines. Results are expressed as the fold of thymidine incorporation in presence of PDGF or FBS over basal unstimulated condition and are the mean ± SEM of three independent experiments performed in quadruplicate. *Significantly different at least at *P* < 0.05 as determined by Student's *t*-test compared with respective basal condition. (c) Indicated serum growing cells were submitted to DNA laddering assays according to standard procedures described in Materials and Methods. As positive control for apoptosis, primary rat thymocytes in culture were exposed 24 hours to anisomycin (10 *μ*g/mL).

In addition to the effect of overexpressing CKI*γ*2 on cell morphology, we found that fibroblasts overexpressing CKI*γ*2 proliferate at a significant slower rate compared with control fibroblasts (Figure 8(a)). In addition, decreased proliferation appears to correlate with the extent of CKI*γ*2 overexpression. Diminished proliferation in cells overexpressing CKI*γ*2 was further confirmed by decreased incorporation of  ^3^H-thymidine into DNA in response to PDGF, a potent mitogenic factor for fibroblast [32], or serum over a 24-hour period of stimulation (Figure 8(b)). For an unknown reason, incorporation of ^3^H-thymidine in response to PDGF or serum stimulation in fibroblasts overexpressing higher levels of CKI*γ*2 (Z6 and Z23) is often decreased compared with their respective unstimulated basal levels (Z6: Bas 5, 300 ± 196, PDGF 3, 205 ± 103, FBS 4, 098 ± 110; Z23: Bas 8, 071 ± 192, PDGF 3, 672 ± 212, FBS 6, 853 ± 327 cpm). Therefore, to exclude cell death as an important factor contributing to decreased proliferation, all cell lines were subjected to DNA laddering assay (Figure 8(c)) and DAPI staining (data not shown). As a positive control for DNA laddering, we used primary cultured rat thymocytes treated for 24 hours with anisomycin (10 ug/mL). Using both approaches, we established that apoptosis is not responsible for the apparent decrease in proliferation of cells overexpressing CKI*γ*2. In agreement, significant increase in doubling time calculated from growth curves for all aforementioned cell lines overexpressing CKI*γ*2 compared with control fibroblasts suggests that overexpression of CKI*γ*2 increases cell cycle duration (Table 1). To test this hypothesis, we performed FACS analysis to determine the distribution of actively serum growing asynchronized cells stably overexpressing CKI*γ*2 throughout the different phases of the cell cycle. As reported in Table 2, 50% of control fibroblasts mock-transfected were detected in G1 and the remaining cell population was evenly distributed into S and G2 phases (approximately 23%, resp.). In contrast, fibroblasts overexpressing CKI*γ*2 presented a significant larger population of cells in G1 (63–70%) and a reduced percentage of cells in S and G2 phases (12–17%). Collectively, these results indicate that CKI*γ*2 inhibits cell proliferation by modulating cell cycle progression through G1.

### 3.5. Overexpression of CKI*γ*2 in Fibroblasts Increases Expression of the CDK Inhibitors p21^Cip1^ and p27^Kip1^ and the Tumor Suppressor p53

**Figure 9:**
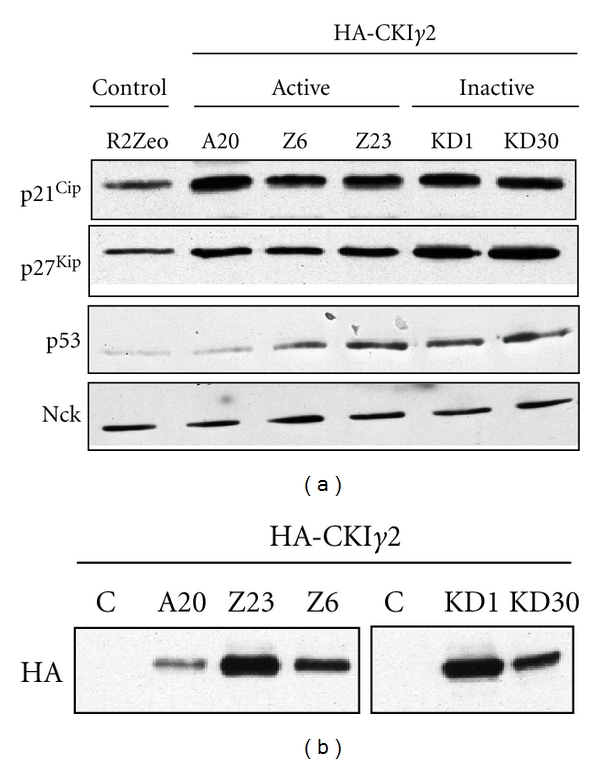
Effect of CKI*γ*2 overexpression on the CDK inhibitors p21^Cip1^ and p27^Kip1^ and the tumor suppressor p53 expression levels. (a) From indicated cell lines, equivalent amount of total cell lysate proteins was subjected to Western Blot analysis using specific antibodies against p21^Cip1^, p27^Kip1^, and p53. Nck Western Blot was used as loading control. (b) Similar samples were probed by western blot with anti-HA antibodies.

Consistent with a larger population of cells in G1 and reduced thymidine incorporation into DNA during the S phase of the cell cycle, earlier G1 phase cell cycle events could account for the antiproliferative effect of CKI*γ*2. To address this, we then compared the expression of the CDKIs p21^Cip1^ and p27^Kip1^ and the tumor suppressor p53 in fibroblasts overexpressing CKI*γ*2 with control fibroblasts. Our investigation revealed that inhibition of cell proliferation and delay in cell cycle progression in fibroblasts overexpressing CKI*γ*2 correlate with increased expression of p21^Cip1^, p27^Kip1^, and p53 (Figure 9(a)). Surprisingly, the effects of CKI*γ*2 on cell cycle regulators are independent of its catalytic activity as shown in fibroblasts overexpressing CKI*γ*2 kinase dead (KD1 and 30) (Figure 3) that still shows increased expression of p21^Cip1^, p27^Kip1^ and p53 proteins. This is in contrast with the effects of CKI*γ*2 on actin reorganization that require the catalytic activity of CKI*γ*2 (Figure 3). Interestingly, increased expression of p21^Cip1^ and p27^Kip1^ appear, to be CKI*γ*2 dosage independent compared to increased expression of p53 which correlates with the levels of CKI*γ*2 overexpressed (Figures 9(a) and 9(b)). Overall, these findings demonstrate that CKI*γ*2 impairs cell proliferation by delaying cells in the G1 phase of the cell cycle. Likewise, the fact that fibroblasts overexpressing CKI*γ*2 are still evenly distributed in S and G2 phases of the cell cycle suggests that these steps proceed normally and that the effects of CKI*γ*2 on cell proliferation are restricted to the G1 phase of the cell cycle.

**Figure 10:**
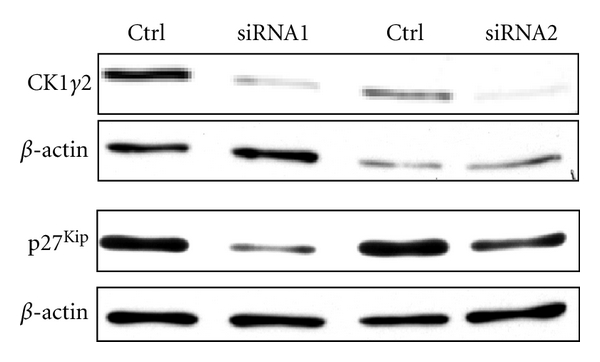
Downregulation of CKI*γ*2 in HepG2 cells decreases expression of p27^Kip1^. HepG2 cells were transiently transfected with control or hCKI*γ*2 (R1, R2) siRNA. Forth eight hours after transfection, cell lysates normalized for protein content were subjected to Western Blot analysis with indicated antibodies.

 To further demonstrate a role for CKI*γ*2 on expression levels of CDK inhibitors, we compared p27^Kip1^ protein expression levels between HepG2 cells transfected with CKI*γ*2 specific siRNAs and scramble siRNA (Figure 10). Using this approach, we found that efficient downregulation of CKI*γ*2 in HepG2 cells leads to decreased expression of p27^Kip1^ proteins.

**Figure 11:**
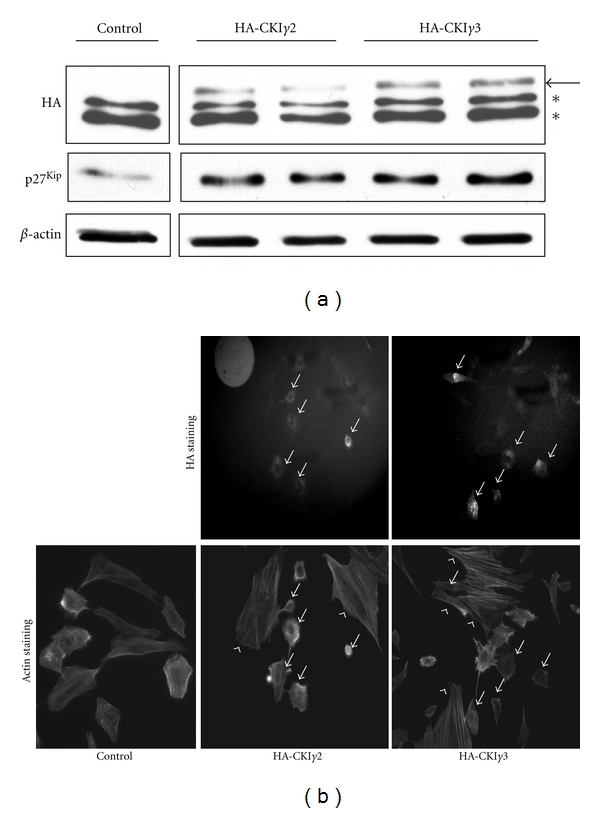
Overexpression of either CKI*γ*2 or CKI*γ*3 in fibroblasts increases expression of p27^Kip1^ and inhibits formation of actin stress fibers. (a) Fibroblasts transiently transfected with plasmids encoding either HA-CKI*γ*2 or HA-CKI*γ*3 were analyzed for HA-tagged proteins and p27^Kip1^ protein expression levels by Western Blot. The arrow represents HA-CKI*γ*2 or HA-CKI*γ*3, while nonspecific bands are indicated by asterisks. Actin was used as loading control. (b) Similar fibroblasts were stained for HA or actin organization using phalloidin coupled to AlexaFluor 488. Arrows indicate HA-positive cells, while arrow heads point nontransfected cells. Pictures were taken at 63X.

CKI*γ*2 is closely related to CKI*γ*1 and 3, and, like CKI *γ*2, CKI*γ*1 and 3 are believed to also be membrane associated due to a putative palmitoylation site present in their C-terminus [25]. In attempt to determine to what extend the effects of CKI*γ*2 on p27^Kip1^ and actin stress fiber are isoform specific, we failed to establish stable fibroblast cell lines overexpressing CKI*γ*1 or 3, most likely due to toxicity as reported by others [33]. This was also the case for transient overexpression of CKI*γ*1 in fibroblasts, while transient overexpression of CKI*γ*2 or *γ*3 was possible. Therefore, we carried out transient transfection of fibroblasts with an empty plasmid as control, or plasmid encoding either HA-tagged CKI*γ*2 or *γ*3 and monitored p27^Kip1^ levels and actin organization using these cells (Figure 11). As reported above, expression of HA-tagged CKI*γ*2 or *γ*3 was detected using total cell lysates in Western Blot with anti-HA antibody (Figure 11(a)). Interestingly, as observed in stable cell lines overexpressing CKI*γ*2, transient overexpression of CKI*γ*2 increases p27^Kip1^ protein levels. However, this is also observed in fibroblasts overexpressing CKI*γ*3 (Figure 11(a)). More importantly, transient overexpression of either CKI*γ*2 or CKI*γ*3 negatively impacts actin stress fibers formation (Figure 11(b)). These results suggest that, like CKI2, CKI*γ*3 could also regulate the expression of CDK inhibitors and actin cytoskeleton reorganization, at least when overexpressed.

### 3.6. Inhibition of Roscovitine-Sensitive Cyclin-Dependent Kinases Reduces the Level of p27^Kip1^ and Rescues Actin Stress Fibers Formation in Fibroblasts Overexpressing CKI*γ*2

**Figure 12:**
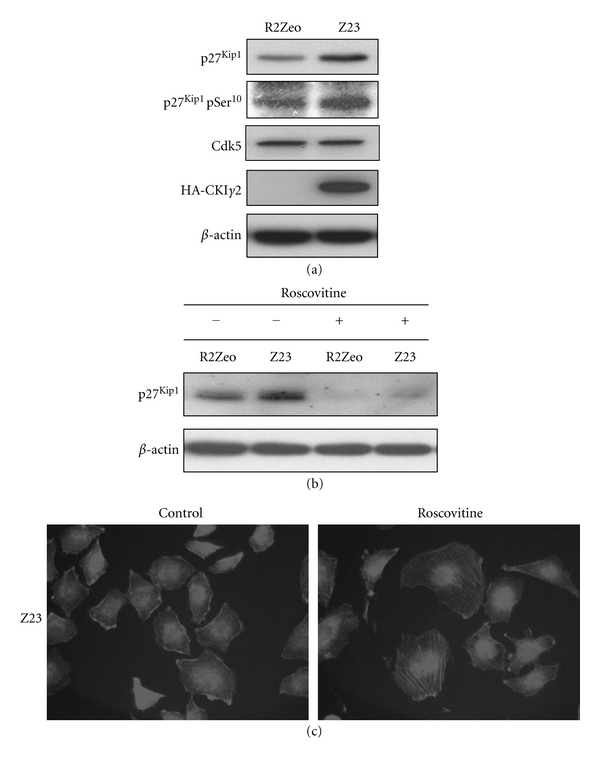
Roscovitine reverses the effects of CKI*γ*2 overexpression on p27^Kip1^ and actin stress fibers. (a) From indicated cell lines, equivalent amount of total cell lysate proteins was subjected to Western Blot analysis using specific antibodies against total and phospho-Ser^10^  p27^Kip1^, Cdk5, and HA. *β*-Actin was used as loading control. (b) Equivalent amount of total cell lysate proteins from indicated cells treated or not with roscovitine that were subjected to Western Blot analysis using specific antibodies against p27^Kip1^ or *β*-actin was used as loading control. (c) Cells overexpressing CKI*γ*2 were incubated with roscovitine (25 *μ*M, 16 hrs) before to be stained with phalloidin to visualized actin organization. Images were taken at 63X.

Since phosphorylation of p27^Kip1^ at Ser10 increases its stability and cytoplasmic accumulation [11, 12] where it can bind and inhibit RhoA [10], we first determined whether p27^Kip1^ phosphorylation at Ser10 was increased in fibroblasts overexpressing CKI*γ*2. Indeed, we found that the level of p27^Kip1^ phosphorylated at Ser10 was higher in CKI*γ*2 overexpressing than in control fibroblasts (Figure 12(a)). In addition, we found that roscovitine, a potent inhibitor of cyclin-dependent kinases with good selectivity toward Cdk1, Cck2, Cdk5, Cdk7, and Cdk9 [34], not only strongly reduced the levels of p27^Kip1^ proteins (Figure 12(b)), but also rescued actin stress fibers formation in fibroblasts overexpressing CKI*γ*2 (Figure 12(c)). Interestingly, we observed that Cdk5, a roscovitine sensitive cyclin-dependent kinase that is phosphorylated and activated by CKI [35–37] and known to affect actin dynamics by interacting and phosphorylating p27^Kip1^ at Ser^10^ [13], is equally expressed in fibroblasts independently of CKI*γ*2 expression levels (Figure 12(a)). Collectively our findings indicate an important role for CKI*γ*2 in modulating actin dynamics through a Cdks-  p27^Kip1^ pathway, potentially implicating Cdk5.

## 4. Discussion

**Figure 13:**
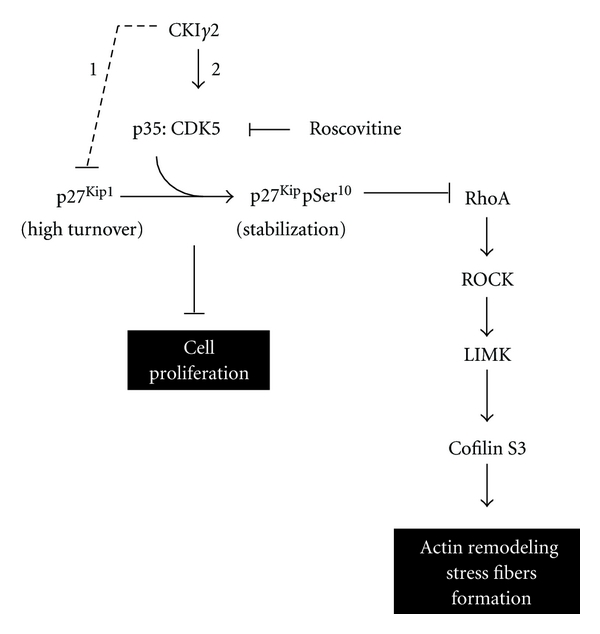
Model describing how CKI*γ*2 prevents the formation of actin stress fibers and regulates cell proliferation. Overexpression of CKI*γ*2 activates Cdk5, which contributes to p27^Kip1^ stabilization and cytoplasmic accumulation following phosphorylation of p27^Kip1^ at Ser^10^. Increased cytoplasmic level of p27^Kip1^ correlates with decreased cellular levels of RhoA. p27^Kip1^ also inhibits RhoA activation by directly binding to RhoA and competing RhoA interaction with RhoGEFs. Reduced RhoA signaling then results in decreased formation of stress fibers. Roscovitine rescues RhoA activation and signaling by inhibiting CKI*γ*2-induced activation of Cdk5 therefore prevents p27^Kip1^ phosphorylation at Ser^10^ and promotes p27^Kip1^ degradation. (1) indicates CKI*γ*2 kinase-independent, while (2) represents CKI*γ*2 kinase-dependent.

 In this study, we provide evidence that the isoform *γ*2 of CKI prevents the formation of actin stress fibers and delays cell cycle progression in G1. We showed that CKI*γ*2 induces phosphorylation and accumulation of p27^Kip1^ and decreases expression levels of RhoA, which could result in inadequate levels of activated RhoA to sustain actin stress fibers formation in fibroblasts expressing higher levels of CKI*γ*2. Moreover, we demonstrate that the effects of CKI*γ*2 on p27^Kip1^ and actin stress fibers are dependent on a subset of Cdks. The findings that CKI regulates Cdk5 activity [35–37] and that Cdk5 is expressed in fibroblasts suggest that the effects of CKI*γ*2 on actin dynamics in fibroblasts overexpressing CKI*γ*2 potentially implicate activation of Cdk5. Several studies indicated that Cdk5 affects actin remodeling in neuronal cells [13, 38–41]. In addition, recent evidence point to a critical role of Cdk5 in the regulation of p27^Kip1^ stability and cytoplasmic retention by directly phosphorylating p27^Kip1^ on Ser10 [13]. Interestingly, a role for p27^Kip1^ in the regulation of RhoA activation [10] has been reported. Indeed, p27^Kip1^ directly interacts with RhoA, inhibiting RhoA activation by interfering with RhoGEFs. Therefore, these findings are consistent with our model suggesting that CKI*γ*2 regulates actin remodeling through a Cdk5-p27^Kip1^-RhoA pathway (Figure 13).

The yeast homologs of the mammalian CKI*γ* isoforms (Yck1/2, Cki1^+^/2^+^) [26] have been implicated in various biological functions. In *S. cerevisiae*, independent loss of function of the *YCK1* and *YCK2* genes did not alter growth, but simultaneous loss of function of both genes resulted in lethality [42]. This established the *YCK* genes as an essential genes pair. In contrast, in *S. pombe*, gene disruption experiments showed that neither cki1^+^ nor cki2^+^ is essential for cell viability [43]. However, overexpression of cki2^+^, but not cki1^+^, resulted in growth inhibition accompanied by aberrant morphology. This suggests that, despite overall similarity in structure, high homology in amino acids sequence and probable overlap in substrate specificity, close related isoforms might have non overlapping functions and play distinct role in cells.

 In this study, we showed that CKI*γ*2 stably overexpressed in fibroblast, alters cell morphology and formation of actin stress fibers concomitant with lower levels of activated RhoA, a small GTPase that regulates actin stress fibers formation in response to growth factors [3]. Interestingly, actin stress fibers were restored by directly activating RhoA signaling following LPA treatment or expression of a constitutively active RhoA, suggesting that CKI*γ*2 regulates upstream events leading to RhoA expression and activation. Meanwhile, we also found that CKI*γ*2 increases expression of the tumor suppressor p53 and the CDK inhibitors p21^Cip1^ and p27^Kip1^ and negatively regulates cell proliferation by delaying cell progression through G1. To explain poor proliferation of CKI*γ*2 overexpressing fibroblasts, we propose that level of RhoA activity in these cells is too low to efficiently counteract the induction of the CDK inhibitors and promote adequate timing of expression of the cyclin D1, both processes normally under the control of RhoA [44–46]. Interestingly, Cdk5 activation in neuronal cells occurs only in postmitotic neurons [47], suggesting that, in fibroblasts overexpressing CKI*γ*2, modulation of the cell cycle resulting in decreased mitotic activity may precede and be required for the activation of Cdk5 by CKI*γ*2. Although additional experiments are required to investigate this point, here we propose a model in which CKI*γ*2 induces the activation of Cdk5 in a kinase-dependent manner to promote cytoplasmic accumulation of the CDK inhibitor p27^Kip1^ that prevents RhoA activation and leads to inhibition of actin stress fibers formation (Figure 13). In summary, this study contributes to improve our knowledge of molecular mechanisms regulating the activity of critical proteins governing actin cytoskeleton dynamics.

## Figures and Tables

**Table 1:** Calculated doubling time from cell proliferation assays.

Clones	Doubling time (hours)
Control R2Zeo	17.3 (0.3)
HA-CKI-*γ*2-Wild Type	
A20	18.8 (0.4)*
Z6	23.1 (0.5)*
Z23	27.1 (0.3)*

Values are the means (SEM) of assays.*P at least ≤ 0.005 compared to control.

**Table 2:** Cell distribution in different phases of cell cycle determined by FACS analysis.

Clones	% fluorescence at cell cycle phase		
	G1	S	G2
Control R2Zeo	50.3 (0.2)	23.6 (0.2)	23.8 (0.1)
HA-CKI-*γ*2-Wild Type			
Z6	67.6 (0.4)*	12.3 (0.4)*	15.3 (0.6)*
Z23	70.2 (0.3)*	17.0 (0.2)*	12.1 (0.4)*

Cells were fixed with 70% ethanol, stained with propidium iodide, and subjected to flow cytometry analysis. Values are mean (SEM) of 4 assays. *P at least ≤ 0.00001 compared to control.
